# RettDb: the Rett syndrome omics database to navigate the Rett syndrome genomic landscape

**DOI:** 10.1093/database/baae109

**Published:** 2024-10-16

**Authors:** Nico Cillari, Giuseppe Neri, Nadia Pisanti, Paolo Milazzo, Ugo Borello

**Affiliations:** Unit of Cell and Developmental Biology, Department of Biology, University of Pisa, S.S.12 Abetone e Brennero 4, Pisa 56127, Italy; Unit of Cell and Developmental Biology, Department of Biology, University of Pisa, S.S.12 Abetone e Brennero 4, Pisa 56127, Italy; Department of Computer Science, University of Pisa, Largo B. Pontecorvo 3, Pisa 56127, Italy; Department of Computer Science, University of Pisa, Largo B. Pontecorvo 3, Pisa 56127, Italy; Unit of Cell and Developmental Biology, Department of Biology, University of Pisa, S.S.12 Abetone e Brennero 4, Pisa 56127, Italy

## Abstract

Rett syndrome (RTT) is a neurodevelopmental disorder occurring almost exclusively in females and leading to a variety of impairments and disabilities from mild to severe. In >95% cases, RTT is due to mutations in the X-linked gene *MECP2*, but the molecular mechanisms determining RTT are unknown at present, and the complexity of the system is challenging. To facilitate and provide guidance to the unraveling of those mechanisms, we developed a database resource for the visualization and analysis of the genomic landscape in the context of wild-type or mutated *Mecp2* gene in the mouse model. Our resource allows for the exploration of differential dynamics of gene expression and the prediction of new potential MECP2 target genes to decipher the RTT disorder molecular mechanisms.

**Database URL**: https://biomedinfo.di.unipi.it/rett-database/

## Introduction

Rett syndrome (RTT) (Online Mendelian Inheritance in Man, OMIM: 312750; Orphanet rare disease code, ORPHA: 778) is a rare neurodevelopmental disorder occurring almost exclusively in females with a frequency of 1 in 10 000 live female births [1]. It is the second cause of severe intellectual disability in females after the Down syndrome [2].

More than 95% of typical RTT cases show mutations in *MECP2* (methyl-CpG binding protein 2) gene [3, 4]. A similar disorder with clinical features of classic RTT but with earlier onset is the congenital variant of this syndrome (OMIM: 613454, ORPHA: 778), caused by mutation in the FOXG1 gene [5]. This pathology and that caused by CDKL5 mutations nowadays are no longer considered atypical RTT cases; they are called, in fact, FOXG1 syndrome or CDKL5 deficiency disorder, respectively.

*MECP2* is an X-linked gene (Xq28) coding for a methyl-CpG binding protein (ENTREZID: 4204), which is a chromatin binding protein [6] expressed ubiquitously but with higher expression in the central nervous system (CNS) [7]. The absence or misfunction of MECP2 results in broad phenotypes including neurological symptoms caused by an incomplete process of neuron maturation and maintenance of fully functional neurons in the CNS [8–12]. While females carrying *MECP2* mutations show a wide spectrum of symptom severities, males generally present severe neonatal encephalopathy with rapid progression (OMIM: 300673) and reduced life expectancy in childhood without an aggressive medical support [13]. However, there have been reports describing boys with nonspecific mental retardation or with less severe neurological and psychiatric manifestations [14–18]; in those cases, the pathological alteration in *MECP2* was associated with X-chromosome aneuploidy or detectable levels of somatic mosaicism [19, 20].

Murine *Mecp2* gene was cloned in 1992, and it was shown to code for a 486-amino-acid-long protein with binding affinity for methylated CpG dinucleotides *in vitro* [6, 21] and characterized as a transcriptional repressor [22]. However, due to the lack of direct experimental evidences, MECP2 has been proposed to repress, activate, or anyway modulate gene transcription [23].

The main function of MECP2 is reading DNA methylation [21, 24, 25]. It has been proposed that MECP2 plays a role in chromatin structure and architecture [26–30], modulating genomic dynamics and formation of nuclear compartments in normal and pathological conditions [31–33]. In addition, MECP2 has been implicated in mRNA splicing [34, 35], miRNA biogenesis [36–39], and long noncoding RNA (lncRNA) activity [40, 41].

Although MECP2 is considered an intrinsically disordered protein [42–44], several distinct structural and functional domains can be recognized at the secondary and the tertiary structure levels: the N-terminal domain (NTD), the methyl-CpG binding domain (MBD), the intervening domain (ID), the transcription repression domain with the nuclear localization signal, the NCoR interaction domain mediating the interaction with the NCoR1/2 corepressor complex, and the C-terminal domain (CTD) [45, 46].

RTT can arise from a series of different *MECP2* mutations causing a range of phenotypes with different degrees of severity [4]. The majority of *MECP2* mutations are localized in the MBD domain, while others affect the NTD or the rest of the protein. Because the MBD domain is the only structured region of the protein and the DNA-binding domain [47], mutations localized here likely change the tertiary structure of the protein and therefore MECP2 binding to DNA and hence the relevance of the MBD mutations.

Human *MECP2* mutations have been reproduced in mouse lines to model the human pathology and to confirm MECP2 involvement in RTT etiology [48]. These mouse models offer the opportunity to study the progression of the pathology from the early stages of brain development to adulthood. These models show a correlation between MECP2 chromatin binding affinity and severity of the phenotype produced by missense mutations. Indeed, mutation R106W (arginine 106 to tryptophan) highly reduces the affinity for the protein to methylated DNA and causes a severe phenotype in humans [49], while mutation T158M (threonine 158 to methionine), which reduces the binding affinity by a much lesser amount than the R106W mutation, determines a less severe phenotype [49, 50]. Confirming this trend, the mildest phenotype is associated with R133C mutation (arginine 133 to cysteine) of MECP2 which binds DNA with similar affinity as the wild-type (WT) protein [51].

Even though mutations in *MECP2* are the major determinants of RTT, the molecular mechanisms linking MECP2 misfunction to the RTT phenotype are still unknown. This is primarily related to the different roles of MECP2 in genome regulation, as discussed earlier. Therefore, at present, it is not possible to design a therapeutic strategy aiming at treating the molecular causes of RTT syndrome via restoration of MECP2 functions. Different clinical trials have been conducted to intervene on specific systems altered in this pathology, but their outcomes are limited to beneficial treatments as supportive care interventions [52]. At present, this pathology is without a cure.

To make sense of the MECP2 regulatory network, integrative analysis of genome-wide data obtained in different models at different levels of gene regulation (i.e. RNA, protein, etc.) is a potent strategy to define specific regulatory or signaling modules [53, 54]. Indeed, given the availability of different types of open access high-throughput expression profiling data, integrative analysis of omics data brings a novel opportunity to model the molecular etiology of neurodevelopmental genetic syndromes like RTT [55]. As detailed later, this powerful integrative approach has been used in modeling the MECP2-regulated gene network, mainly at the level of transcripts and/or proteins, in nonpathological and RTT conditions to identify the molecular pathways dysregulated in the RTT CNS neurons.

Most analyses were conducted at the level of the transcriptome using different microarray data of RTT and control samples of human and mouse brain tissues as well as neuronal cells. Those analyses aimed at identifying the common elements dysregulated in RTT by a pathway and gene network analysis [56–60]. A similar analysis was performed by Haase *et al*. comparing microarrays with RNA-Seq data of human and mouse RTT models [61].

RTT mouse model cortex has been also the focus of integrative genome-wide analysis of transcriptomic and proteomic datasets with the goal of performing pathway analysis [62, 63].

In addition, Yu *et al*. identified key RTT factors involved in the process of RNA splicing, comparing the RNA-Seq dataset of a mouse RTT model with a comprehensive and curated signature database of RNA-Seq generated after perturbing many splicing factors [64].

In the framework of those analyses, our work offers a different perspective on *Mecp2* gene regulation activity by combining the transcriptome data of a mouse model of *Mecp2* Knock-Out (KO) [65] with the genome-wide MECP2 binding activity and chromatin status profile in WT animals to analyze and visualize MECP2 function as a chromatin-dependent transcriptional regulator.

With the goal of comparing homogeneous data, we analyzed datasets almost exclusively derived from cerebral cortex at 6/8 weeks after birth of a single RTT mouse model [65].

To grant the best accessibility of our integrative analysis, we embedded our results in a Genome Browser easily and freely available to the scientific community.

Our resource provides a powerful tool to unravel RTT molecular mechanisms, allowing for the visualization of transcriptomic changes in the context of the chromatin landscape.

Moreover, because RTT is a monogenic syndrome with a range of typical symptoms of the autism spectrum disorder [16, 66, 67], RettDb also offers a simplified context for studying the molecular mechanisms underlying the etiology of autism.

## Methods

### Genome browser development

The Genome Browser structure is based on the WashU Epigenome Browser [68]. WashU Epigenome Browser, executed without any modification or customization, is embedded in a HTML5 static website that includes a brief description of the RTT, the Genome Browser, references to the data sources, and the information on the research groups involved in this project.

The reference genome for the datasets loaded into the Genome Browser is *Mus musculus* mm10.

The whole website, including the database, is stored in a virtual machine provided by the Green Data Center of the University of Pisa, currently configured with four cores Xeon Gold 5218, 8 GB RAM, 700 GB disk space. The large availability of computational resources allows for the extension of the virtual machine with more cores, RAM, and disk space as needed.

### Data analysis

The raw files (fastq files) of ChIP-Seq and RNA-Seq were aligned to the mouse reference genome (mm10) using Bowtie2 [69] and STAR software [70], respectively, using default parameters. The sources of the datasets used to populate the Genome Browser are listed in Supplementary Table S1.

Peak calling for ChIP-Seq files was performed using MACS v1.4.2 or MACS2 software [71, 72] with default parameters for MECP2 ChIP-Seq data or with the ‘--broad’ and ‘--nomodel’ parameter for histone modifications. The epigenomic marker ChIP-Seq biological replicates from WT animals have been pooled and analyzed together, resulting in a single track. Three different MECP2 ChIP-Seq biological replicates from WT animals (Supplementary Table S1) have been analyzed independently and shown in separate tracks.

Published RNA-Seq count tables of WT and *Mecp2* KO samples (Supplementary Table S1) were used to count the reads mapping on genomic regions annotated as genes in the mouse genome (mm10) using HTseq-count software [73], with parameters--order pos--mode union--stranded yes--type exon--idattr gene_id. Differential genes were called with DESeq2 with default parameters and calling differentially expressed genes (DEGs), those genes with a *P*-value corrected for multiple testing (*P*_adj_) of <.1 [74].

Chromosomal interaction maps of WT and *Mecp2* KO samples, derived from high-throughput chromatin conformation capture (HI-C) experiments, were converted using Juicer [75] to highly compressed binary files (HI-C files) and displayed as separate tracks on the Genome Browser.

Supplementary Table S2 lists all the software packages used for the analyses.

## Results

### RettDb web interface

Our resource was designed to integrate specific RTT omics data in a Genome Browser, allowing for their visual interpretation in the context of the epigenomic WT landscape. To this purpose, we collected different datasets derived from the Bird’s KO *Mecp2* mouse model [65, 76] (Supplementary Table S1) and loaded them onto the Genome Browser along with the WT datasets [7, 77–80]. To make these datasets comparable, we collected data mainly of a single tissue at a single postnatal time point, i.e. the cerebral cortex at 6/8 weeks of postnatal life. In fact, MECP2 seems to play a more functional role during the early postnatal period than during embryogenesis [81, 82].

**Figure 1.**
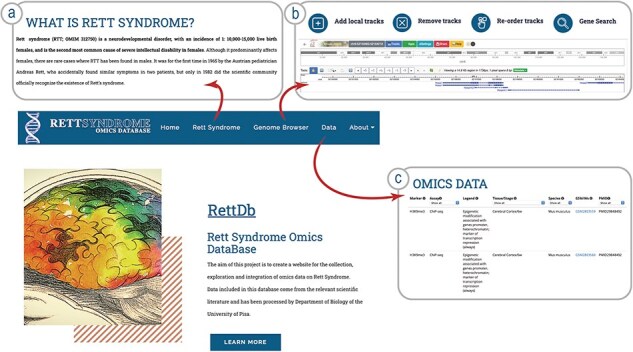
Schema of the RettDb database structure. (a) RTT description page, (b) link to the Genome Browser landing page, and (c) link to the list of datasets loaded onto the Genome Browser.

**Figure 2.**
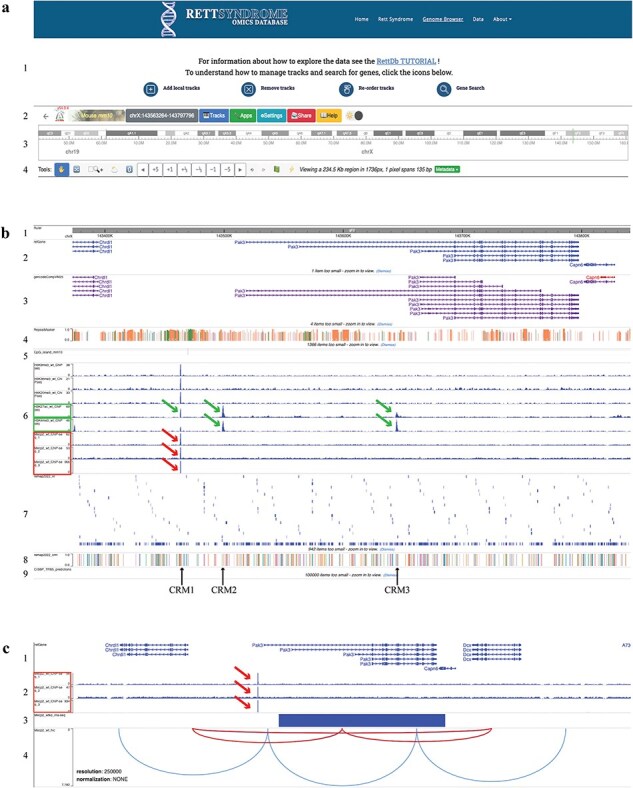
The RettDb database Genome Browser. (a) Genome browser top page: (1) links to tutorials, (2) settings, (3) chromosome ideogram, and (4) genomic view settings. (b) Genome browser tracks: (1) genomic ruler, (2) RefGene gene models, (3) Gencode gene models, (4) repeat masker, (5) CpG islands, (6) histone modifications and MECP2 ChIP-Seq peaks, (7) ReMap NR, (8) ReMap CRM, and (9) CIS-BP. (c) Tracks showing (1) RefGene gene models, (2) MECP2 ChIP-Seq peaks, (3) RNA-Seq track, and (4) HI-C maps. (c) A magnified image of the *Pak3* locus as compared to (b). In this panel, the MECP2 ChIP-Seq tracks are only shown. The green frames in (b) (6) show H3K27ac and H3K4me3 peaks (green arrows), and the red ones show the MECP2 ChIP-Seq peaks (red arrows). The solid blue rectangle in (c) (3) indicates that *Pak3* gene is downregulated in the MECP2 RNA-Seq data. Black arrows indicate the ReMap CRM positions. The red arcs in (c) (4) indicate the chromatin loops that include the putative *Pak3* enhancer where MECP2 binds and the *Pak3* locus.

The resource we describe here is a Genome Browser freely accessible to the scientific community via a web interface. The web tool landing page contains a general description of the resource (Fig. 1). The banner on the top contains links to the other pages. The ‘Rett Syndrome’ page briefly describes the clinical features of the RTT (Fig. 1a); the ‘Genome Browser’ link opens our Genome Browser landing page, from which is possible to access the different tracks (Figs. 1b and 2).

The ‘Data’ page (Fig. 1c) contains information regarding how to obtain the data integrated in the RettDb (with a link to the dedicated page on Zenodo) and how to execute a local instance of RettDb through a Docker container developed on purpose. Moreover, the page contains a table (Supplementary Table S1) with information about the original datasets used, described as follows: (i) marker shown in the tracks, (ii) type of assay used to obtain the results, (iii) description of the data, (iv) source and postnatal stage of the tissue used in the experiment, (v) species, (vi) Dataset (GEO ID: GSM/GSW) of the samples, and (vii) PMID of the publications describing the experiments.

### Data visualization with the Genome Browser

The core of our resource is the Genome Browser (Fig. 2), accessible from the ‘Genome Browser’ link from the landing page, as already described (Fig. 1b).

The text paragraph at the top of this page contains a link to a step-by-step tutorial that guides the user into the RettDb functionalities. The icons located below the banner open tutorials on how to add and remove local tracks, reorder them, and search for a specific gene (Fig. 2a1).

The top menu bar contains tools included in the original WashU Epigenome Browser architecture and allows the user to change most of the browser functionality (Fig. 2a2). Moving from the right to the left, there are (1) the browser logo showing the browser version information; (2) the reference genome (here set to *Mus musculus* mm10); (3) the genomic position icon, a convenient dropdown menu to set the Genome Browser on a specific genomic position (see below); (4) the Tracks menu, a dropdown menu with the track list and tracks tools; (5) the Apps menu, a series of tools useful for the visualization and analysis of the genomic region of interest; (6) the Settings menu; (7) a sharing data tool; and (8) the link to the WashU Epigenome Browser original documentation.

To select a specific genomic region, the genomic coordinates of the region of interest can be changed in the ‘Region search’ box of the genomic position icon. From this dropdown menu, it is also possible to search for a specific gene by its gene symbol in the ‘Gene search’ box or to search for a specific single-nucleotide polymorphism (SNP) by its ID in the ‘SNP search’ box.

The chromosome ideogram is shown in Fig. 2a3; other tools allow us to change the position and the size of the genomic region of interest (Fig. 2a4).

The first genomic tracks under the genome ruler (Fig. 2b1) are the ideograms of the RefGene [83] and Gencode [84] gene models, which are showed together with the repeat masker track (Fig. 2b2–4). The CpG islands track (Fig. 2b5) helps identifying transcription start sites and promoter regions.

The ChIP-Seq tracks, obtained from WT animals’ datasets (Supplementary Table S1), show genome-wide MECP2 binding sites overlapping to putative regulatory regions in the context of a specific epigenomic landscape (Fig. 2b6). The user can visualize different epigenetic modification peaks by selecting specific tracks as described in ‘Add Local tracks’ (Fig.2a1) to further contextualize MECP2 genome-wide binding within chromatin status.

To identify transcription factors (TFs) potentially interacting with MECP2 on genomic regulatory elements, we incorporated in the database the nonredundant (NR) peaks and *Cis* Regulatory Module (CRM) data of the ReMap project (Fig. 2b7,8). The ReMap catalog consists of manually edited, high-quality regulatory regions selected from a large-scale analysis of DNA-binding experiments [85]. Clicking on the ReMap NR rectangles, hover boxes will show the TF name, the cellular and tissue type from which the data were obtained, and the genomic coordinates of the binding site. The CRM track shows the first of the many TFs binding on that regulatory region and the genomic coordinates of the CRM. For the whole list of TFs, the user is referred to the ReMap database, using the genomic coordinates indicated in the hover box.

In addition, we provide a track showing putative TF DNA-binding motifs obtained from the mouse Catalog of Inferred Sequence Binding Preferences (CIS-BP) [86] (Fig. 2b9). Clicking on CIS-BP rectangles, hover boxes will show the TF name and the genomic coordinates of the binding sites.

The potential MECP2 target genes can be identified using the track showing the DEGs derived from the RNA-Seq dataset obtained comparing *Mecp2* KO to WT gene expression (Fig. 2c3). In this track, the upregulated or downregulated gene is indicated by a red or blue rectangle, respectively; the rectangle corresponds to the gene model indicated in the RefGene track (Fig. 2b2 and c1).

Data integration within the Genome Browser allows the identification of MECP2 direct and indirect target genes; the direct target genes are further identified by the presence of MECP2 ChIP-Seq peaks on their regulatory regions identified by the ReMap and the epigenomic data (Fig. 2b6–8 and 2c2).

Moreover, the HI-C experiments identify topologically associating chromatin domains (TADs). TADs represent genomic *cis*-interactions where enhancers contact promoters more frequently in a specific TAD than outside [87]. We show functional chromatin loops, identified by TADs, because they couple specific regulatory regions to their target genes in the context of WT and KO *Mecp2* samples (Fig. 2c4).

Finally, right clicking on all the tracks’ name opens a dialogue box with the track data visualization settings.

A step-by-step tutorial is available on the Genome Browser page to guide the user into the RettDb functionalities.

### Case study

To demonstrate the usefulness of our resource, we searched for novel putative MECP2 targets. Specifically, we focused on PAK3, a downstream effector of the Rho family of GTPases, and a p21-activated serine/threonine kinase involved in cytoskeletal remodeling and spine morphogenesis [88]. The rationale of this choice is based on our previous experimental evidences, demonstrating that downregulation of *Pak3* blocks neurite growth in immature interneurons [89] and that *Mecp2* KO models showed dendritic spine density reduction in cortical and hippocampal neurons [82, 90, 91]. Importantly, *PAK3* misregulation is related to X-linked cognitive disability phenotypes [92, 93].

When we analyzed the *Pak3* locus, we observed a MECP2 binding peak on a putative regulatory region upstream of one of the *Pak3* transcription start sites (Fig. 2b6–c2, red arrow). This site overlaps with the H3K27ac peak, an epigenetic marker for active enhancer (Fig. 2b6, green arrows) and one of the CRMs (CRM1) identified by the ReMap project (Fig. 2b8, black arrow). These findings are consistent with the localization of a MECP2 binding site in a putative *Pak3* enhancer. Peaks of epigenetic markers of repressed chromatin state are also present at this position (Fig. 2b6) even though at lower reads density, suggesting a more complex and dynamic regulation. These data suggest that *Pak3* is a MECP2 direct target. Specifically, MECP2 may positively regulate *Pak3* expression, as further suggested by the decreased expression of *Pak3* in the *Mecp2* KO compared to WT, as shown in the MECP2 RNA-Seq track (Fig. 2c3, blue rectangle).

H3K27ac and H3K4me3 peaks are also located at the 5ʹ end of different *Pak3* isoforms, indicating active transcription of *Pak3* locus (Fig. 2b6, green arrows); these peaks overlap with other CRMs (Fig. 2b8, CRM2 and CRM3 indicated by black arrows). Although these ChIP-Seq datasets do not show MECP2 peaks in CRM2 and CRM3, MECP2 binding in these regions is predicted by the presence of a putative MECP2 binding consensus sequence (Table 1). Moreover, when we analyzed the list of the TFs binding to the CRMs, obtained from the ReMap and CIS-BP data, we noticed that the majority of those TFs are differentially expressed in *Mecp2* KO vs WT cerebral cortex (Table 1; DEGs identified in the RNA-Seq dataset track as in Fig. 2c3 for *Pak3*), suggesting that they are either direct or indirect MECP2 targets.

**Table 1.** List of the TFs binding to the CRMs identified by ReMap data

**CRM 1**	DEGs	Aebp2, Aff4, Ar, Arid1a, Arntl, Asc1l, Ascl2, Ash2l, Atf3, Atf4, Atf7, Atf7ip, Atoh1, Atrx, Bach1, Bach2, Batf, Batf3, Bbx, Bcl11b, Bcl6, Bhlha15, Bhlhe40, Bhlhe41, Brca1, Brd2, Brd3, Brd4, Brd9, Cbfb, Cbx2, Cbx5, Cbx7, Cdk9, Cebpa, Cebpb, Cebpg, Chd4, Chd7, Chd8, Clock, Creb1, Creb3l2, Crebbp, Crtc2, Cry1, Ctcf, Ctnnb1, Cux2, Ddit3, Dnmt3a, Dnmt3b, E2f3, E2fl, Ebf1, Ebf2, Eed, Egr2, ELf1, Elf5, Elk1, Ep300, Ep400, Epop, Erf, Erg, Esr1, Esrrb, Ets1, Ets2, Etv6, Ezh1, Ezh2, Fgfr1, Fli1, Fos, Fosl2, Foxa2, Foxh1, Foxj2, Foxo1, Foxo3, Foxp1, Foxp3, Gabpa, Gata2, Gata3, Gata4, Gata6, Gfi1, Gli1, Gps2, Grhl3, Grip1, Gtf2b, Gtf2e, Gtf3c1, Hand1, Hand2, Hdac1, Hdac2, Hdac3, Hdac4, Hes1, Hes5, Hey1, Hey2, Hif1a, Ikzf1, Irf1, Irf3, Irf4, Irf8, Irf9, Isl1, Jarid2, Jmjd6, Jun, Junb, Jund, Kat2a, Kat2b, Kdm3a, Kdm4a, Kdm4c, Kdm6b, Kdma1a, Klf3, Klf4, Klf5, Kmt2a, Kmt2b, Kmt2d, Ldb1, Lhx2, Lhx3, Lmnb1, Lmx1b, Maf, Mafa, Mbd1, Mbd2, Mbd3, Mbd4, Mecp2, Med1, Med12, Med23, Med26, Mef2a, Meis1, Men1, Mettl3, Mtf2, Myb, Myc, Myod1, Myog, Nanog, Ncaph2, Ncoa2, Ncoa3, Ncor1, Ncor2, Nelfe, Neurod1, Neurod2, Nfat5, Nfatc1, Nfe2, Nfib, Nfic, Nfil3, Nfya, Nipbl, Nkx2-1, Nkx2-2, Npas4, Nr1d1, Nr1d2, Nr1h4, Nr3cl, Nr4a1, Nr5a2, Olig2, Otx2, Pax3, Pax5, Pax7, Pbx1, Pcgf2, Pcgf3, Pcgf5, Pcgf6, Pcgfl, Pgr, Phf5a, Phf8, Pml, Pou3f2, Pou5f1, Ppara, Pparg, Ppargc1a, Prdm1, Prdm13, Prdm16, Prdm5, Prox1, Rad21, Rad51, Rar, Rara, Rarb, Rbp1, Rbpj, Rcor1, Rela, Rest, Rfx1, Rnf2, Rora, Rorc, Rpa1, Rsf1, Runx1, Runx2, Runx3, Runxltl, Rxra, Sall4, Satb1, Setdb1, Shox2, Sin3a, Sinhcaf, Smad2, Smad3, Smad4, Smarca4, Smarca5, Smarcc1, Smc1a, Smc3, Snai2, Snail, Sox17, Sox2, Sox3, Sox5, Sox6, Sox9, Sp1, Sp7, Spi1, Spib, Srebfl, Srf, Ssl8, Ssrp1, Stag1, Stag2, Stat1, Stat2, Stat3, Stat4, Stat5a, Stat5b, Stat6, Supt5, Suz12, T, Taf3, Tafl, Tal1, Tbl1x, Tbp, Tbx19, Tbx21, Tbx6, Tcf3, Tcf4, Tcf7, Tcf71l, Tcf7l2, Tead2, Tead4, Terf2, Terf2ip, Tet2, Thap11, Thrb, Tle3, Top2a, Trim28, Trp53, Trp73, Trps1, Twist1, Usf1, Vdr, Wdr5, Xbp1, Yap1, Yy1, Zbtb16, Zbtb17, Zfp143, Zfp296, Zfp809
	N-DEGs	Atf2, Ctcfl, Dmc, Foxa1, Foxd3, Gata1, Gtf2a, Gtf2f, Gtf2h, Hnf1a, Hnf4a, Hoxc10, Ikzf, Isl1-2, Isl1-lhx3, Myf5, Nkx31, Nr1h2-3, Phox2b, Ptf1a, Rf7, Runx1-Eto, Rxr, Rxra-B, Rxra-G, Smad2-3, Stat5, Tead, Ubtf, Zfp683
**CRM 2**	DEGs	Arnt2, Arntl, Ascl1, Ash2l, Atrx, Bhlha15, Brd2, Brd4, Cbx3, Cbx7, Cdk9, Cebpa, Chd4, Creb1, Ctcf, Dmc1, Ep300, Epop, Epop, Erf, Esr1, Esr1, Ets1, Etv6, Ezh2, Fosl1, Fosl2, Foxh1, Gfi1, Gtf2b, Hdac1, Hdac2, Hdac3, Hey2, Ikzf1, Irf4, Irf8, Jarid2, Jarid2, Kdm1a, Kdm4a, Kdm4c, Kdm5a, Klf4, Kmt2b, Kmt2d, Lhx2, Lmo2, Mbd2, Mecp2, Med1, Med1, Men1, Mtf2, Myc, Myod1, Myog, Myt11, Ncor2, Nipbl, Nipbl, Nr3cl, Nr3cl, Pax5, Pax6, Pax7, Pbx1, Pcgf1, Pcgf6, Pgr, Phf19, Rad21, Rail, Rbfox2, Rela, Rnf2, Runx2, Runx3, Shox2, Smad3, Smarca4, Smarca5, Smc1a, Snai12, Sox2, Sox2, Sox4, Sp1, Sp3, Sp7, Spi1, Srf, Srf, Stag1, Stat1, Stat3, Supt16, Suz12, Suz12, Tbl1x, Tcf12, Tcf3, Tead1, Terf2, Tet1, Tet2, Top2a, Top2b, Trim28, Twist2, Xbp1, Yy1, Zbtb11, Zbtb17
	N-DEGs	Ascl, Bhlhe40, Ctcfl, Dppa4, Myf5, Pgr, Pou5f1
**CRM 3**	DEGs	Ascl1, Ascl2, Ash2l, Asxl1, Atf2, Atf7, Atoh1, Brd2, Brd4, Cbx1, Cbx3, Cbx7, Cebpb, Chd4, Creb1, Crebbp, Ctcf, Dppa4, Ep300, Esr1, Esr2, Ezh1, Ezh2, Fos, Fosb, Fosl2, Foxa2, Foxh1, Gfi1, Gtf3c1, Hcfc1, Hdac1, Hdac2, Hdac3, Hmga2, Ikzf1, Isl12, Jarid2, Jun, Jund, Kdm1a, Kdm3a, Kdm4a, Kdm4c, Kdm5a, Klf3, Klf4, Klf5, Klf9, Kmt2b, Kmt2d, Lmnb1, Mbd2, Mcrs1, **Mecp2**, Med1, Med24, Myc, Myt1l, Ncaph2, Ncor2, Nkx2-1, Nr3c1, Olig2, Pax7, Pcgf6, Pgr, Phip, Prdm1, Prdm13, Rad21, Rai1, Rbfox2, Rfx1, Rnf2, Runx1t1, Runx2, Sap130, Shox2, Sin3a, Sinhcaf, Smad3, Smarca4, Smc1a
	N-DEGs	Cdx2, Dppa4, Pou5f, Runx1-eto

TFs are divided in DEGs and N-DEGs as reported in the MECP2 RNA-Seq dataset. The DEG vs N-DEG expression has been analyzed using the RNA-Seq dataset as for Pak3 in Fig. 2c3. Mecp2 is shown in bold and underlined. CRM1, 2, and 3 positions are indicated by black arrows in Fig. 2b7. Abbreviations: N-DEG, non differentially expressed gene.

Finally, the HI-C experiments in WT samples show two loops, a larger one containing the CRM1, CRM2, CRM3, the *Pak3* promoter, and the gene body and a smaller one containing the CRM1, CRM2, and the *Pak3* promoter (Fig. 2c4). The same loops are present in the KO HI-C experiments, suggesting that MECP2 does not change the structure of those specific loops (data not shown).

In conclusion, our new web database allowed us to identify *Pak3* as a putative MECP2 direct target gene.

## Discussion

We present here a new web resource for the visualization and analysis of *Mecp2* genome-wide gene expression regulation. MECP2 is a reader of the chromatin methylation marks and a regulator of chromatin architecture and gene expression [21, 24–26, 28, 29]. Mutations in *MECP2* are found in patients affected by RTT [3, 4], the second cause of severe intellectual disability in females after the Down syndrome [2].

The aim of this work is to provide the scientific community with a freely available repository of omics datasets related to the RTT syndrome and to MECP2 function in the brain.

Previous integrative studies of omics data analyzed and compared transcriptomic and proteomic datasets in order to identify the specific pathways and processes altered when *Mecp2* is mutated [53–60]. However, MECP2 seems to regulate gene expression by reading and binding to specific chromatin regions [21, 24, 25]. Therefore, we reasoned that a different approach integrating gene expression with epigenomic datasets would be key to fully understand MECP2 functions and RTT molecular mechanisms. In fact, our web resource enables the analysis of changes in gene expression induced by mutated *Mecp2* together with the MECP2 genome-wide binding profile in the context of the WT epigenetic landscape. This perspective provides new insights into MECP2 function as a chromatin-dependent transcriptional regulator and allows for the identification of MECP2 direct and indirect target genes. We focused here on MECP2 function as a chromatin state reader and, therefore, we show histone marker ChIP-Seq datasets from WT samples only. Moreover, to correlate putative enhancers with specific gene expression activities, we visualized loops of functional chromatin using HI-C data. In this case, given the MECP2 role in regulating chromatin architecture [26–30], we used both WT and KO *Mecp2* HI-C datasets.

To show the functionality of our tool, we presented here a case study built searching for novel putative MECP2 targets. *Pak3* is a well-known downstream effector of the Rho family of GTPases and a p21-activated serine/threonine kinase involved in cytoskeletal remodeling and spine morphogenesis [88], processes dysregulated in the RTT neurons [8–10]. We, therefore, tested if *Pak3* is a MECP2 direct target. When we analyzed the expression of *Pak3*, the RNA-Seq track showed that this gene is downregulated in *Mecp2* KO mice. Moreover, on the *Pak3* genomic locus, we identified one regulatory region that could mediate MECP2 direct transcriptional regulation of *Pak3* expression. This region contains a MECP2 binding peak together with peaks of the epigenetic markers of active chromatin within a CRM (CRM1), suggesting that MECP2 may bind a *Pak3* enhancer. This observation is further supported by the HI-C data, showing an arc of functional chromatin containing this putative enhancer together with *Pak3* locus.

In addition, we found additional peaks of active chromatin markers located upstream of different *Pak3* isoforms within the other two CRMs (CRM2 and 3), putative *Pak3* promoters, as suggested by these data. Even though the ChIP-Seq data we analyzed did not show MECP2 binding peaks in the CRM2-3, the presence of putative MECP2 binding sites strongly suggests its involvement in the regulation of *Pak3* via those CRMs. Another important information we obtained with our web resource was that most of the TFs binding CRM1, CRM2, and CRM3 showed altered expression in the *Mecp2* KO mice, suggesting that they may be MECP2 targets as well as coregulators of the MECP2-mediated gene expression activity.

In summary, the combination of transcriptomic and epigenomic data in our web database allowed for the identification of *Pak3* as a likely direct target of MECP2 regulation. These observations, together with the published results showing that *Pak3* misregulation correlates with neuronal morphology defects observed in RTT [8–10, 88], strongly point at PAK3 as a putative MECP2 effector and a possible determinant of the cytoskeletal defects observed in the neurons in RTT patients’ brains.

Our data show the value of our resource to formulate hypothesis, which could be further confirmed experimentally.

In the database we presented here, we used data derived from bulk omics datasets limited to male adult cerebral cortex at 6–8 weeks after birth of a single RTT mouse model. The rational of this choice was to provide the best pool of homogeneous data avoiding confounding factors and to offer a solid starting point for integrating new data, in terms of age, sex, tissue, and cellular source. Indeed, new data are now available, which allows us to study MECP2 function in a cell type–specific perspective [62]. In this context, highly informative data are the transcriptome and epigenome analyzed at the single-cell level, which allows us to obtain information about the effect of MECP2 mutations at cellular- and allele-specific levels [94, 95]. This is particularly important considering that in females, the expression of MECP2 mutation is a mosaic due to random X-chromosome inactivation [96, 97]. Therefore, in the future, we will include in our resource data generated by single-cell experiments to take into account MECP2 mutations in heterozygous females. In addition, we plan to include in the database epigenomic markers ChIP-Seq data of the *Mecp2* KO cortices to visualize MECP2 function not only as a chromatin reader but also as a chromatin modifier. Finally, we aim at expanding this resource with the addition of the human RTT data, providing access to a comprehensive and curated repository of all omic datasets generated in RTT models as well as a list of papers published on the subject. The resource will be provided with a link to bioactive small molecules interacting with key RTT genes and to the published Wiki-Pathway visualization of the genes involved in this pathology [58].

In conclusion, our data highlight the potential of RettDb to provide a useful tool to formulate new hypothesis and uncover new mechanisms of gene expression and regulation. The continuous development of this resource, incorporating new datasets derived from emerging and refined omic techniques [98], will be of paramount importance to improve the predictability of our database in order to shed light on the molecular mechanisms of RTT and the development of new drugs for clinical and preclinical screenings.

## Supplementary Material

baae109_Supp

## Data Availability

All the RettDb data are deposited and freely available on Zenodo open data repository (DOI: 10.5281/zenodo.12819274).
