# Supplementary material for: RettDb: the Rett syndrome omics database to navigate the Rett syndrome genomic landscape
Source: Database (Oxford). 2024 Oct 16;2024:baae109. doi: 10.1093/database/baae109 (PMC11482253; doi:10.1093/database/baae109)
Supplement: baae109_Supp [file baae109_supp.zip › baae109_supp/Sup_Tab1_Cillari.pdf]

Marker	Assay	Legend	Tissue/Stage	Species	Dataset ID	PMID
H3K9me3	ChIP-Seq	Epigenetic modification associated with genes promoter, heterochromatin. Marker of transcription repression.	Cerebral Cortex/ 6 weeks	Mus musculus	GSM2803559	PMID29848492
H3K9me3	ChIP-Seq	Epigenetic modification associated with genes promoter, heterochromatin. Marker of transcription repression.	Cerebral Cortex/ 6 weeks	Mus musculus	GSM2803560	PMID29848492
H4K20me3	ChIP-Seq	Epigenetic modification associated with genes promoter, heterochromatin. Marker of transcription repression.	Cerebral Cortex/ 6 weeks	Mus musculus	GSM2803563	PMID29848492
H4K20me3	ChIP-Seq	Epigenetic modification associated with genes promoter, heterochromatin. Marker of transcription repression.	Cerebral Cortex/ 6 weeks	Mus musculus	GSM2803564	PMID29848492
H3K36me3	ChIP-Seq	Epigenetic modification associated with gene body and transcriptional activation.	Cerebral Cortex/ 6 weeks	Mus musculus	GSM2803551	PMID29848492
H3K36me3	ChIP-Seq	Epigenetic modification associated with gene body and transcriptional activation.	Cerebral Cortex/ 6 weeks	Mus musculus	GSM2803552	PMID29848492
H3K4me3	ChIP-Seq	Epigenetic modification associated with genes promoter, bivalent promoters, TSS and transcriptional activation.	Cerebral Cortex/ 6 weeks	Mus musculus	GSM2803555	PMID29848492
H3K4me3	ChIP-Seq	Epigenetic modification associated with genes promoter, bivalent promoters, TSS and transcriptional activation.	Cerebral Cortex/ 6 weeks	Mus musculus	GSM2803556	PMID29848492
H3K27ac	ChIP-Seq	Epigenetic modification associated with active enhancers and promoters.	Cerebral Cortex/ 7-8 weeks	Mus musculus	GSM3502267	PMID31784360
H3K27ac	ChIP-Seq	Epigenetic modification associated with active enhancers and promoters.	Cerebral Cortex/ 7-8 weeks	Mus musculus	GSM3502273	PMID31784360
H3K27ac	ChIP-Seq	Epigenetic modification associated with active enhancers and promoters.	Cerebral Cortex/ 7-8 weeks	Mus musculus	GSM3502281	PMID31784360
H3K27ac	ChIP-Seq	Epigenetic modification associated with active enhancers and promoters.	Cerebral Cortex/ 7-8 weeks	Mus musculus	GSM3502289	PMID31784360
H3K27ac	ChIP-Seq	Epigenetic modification associated with active enhancers and promoters.	Cerebral Cortex/ 7-8 weeks	Mus musculus	GSM3502293	PMID31784360
H3K27ac	ChIP-Seq	Epigenetic modification associated with active enhancers and promoters.	Cerebral Cortex/ 7-8 weeks	Mus musculus	GSM3502297	PMID31784360

H3K27ac	ChIP-Seq	Epigenetic modification associated with active enhancers and promoters.	Cerebral Cortex/ 7-8 weeks	Mus musculus	GSM3502303	PMID31784360
H3K27ac	ChIP-Seq	Epigenetic modification associated with active enhancers and promoters.	Cerebral Cortex/ 7-8 weeks	Mus musculus	GSM3502305	PMID31784360
H3K27ac	ChIP-Seq	Epigenetic modification associated with active enhancers and promoters.	Cerebral Cortex/ 7-8 weeks	Mus musculus	GSM4114271	PMID31784360
H3K27ac	ChIP-Seq	Epigenetic modification associated with active enhancers and promoters.	Cerebral Cortex/ 7-8 weeks	Mus musculus	GSM4114273	PMID31784360
H3K27ac	ChIP-Seq	Epigenetic modification associated with active enhancers and promoters.	Cerebral Cortex/ 7-8 weeks	Mus musculus	GSM4114275	PMID31784360
H3K27ac	ChIP-Seq	Epigenetic modification associated with active enhancers and promoters.	Cerebral Cortex/ 7-8 weeks	Mus musculus	GSM4114277	PMID31784360
H3K27ac	ChIP-Seq	Epigenetic modification associated with active enhancers and promoters.	Cerebral Cortex/ 7-8 weeks	Mus musculus	GSM4114279	PMID31784360
MECP2	ChIP-Seq	ChIP-Seq performed on MECP2 protein.	Cerebral Cortex/ 6 weeks	Mus musculus	GSM2410973	PMID27965390
MECP2	ChIP-Seq	ChIP-Seq performed on MECP2 protein.	Cerebral Cortex/ 6 weeks	Mus musculus	GSM2410975	PMID27965390
MECP2	ChIP-Seq	ChIP-Seq performed on MECP2 protein.	Cerebral Cortex/ 6 weeks	Mus musculus	GSM2410977	PMID27965390
MECP2	ChIP-Seq	ChIP-Seq performed on MECP2 protein.	Forebrain/ 8 weeks	Mus musculus	GSM1464563	PMID25762136
MECP2	ChIP-Seq	ChIP-Seq performed on MECP2 protein.	Brain Tissue/ 6-8 weeks	Mus musculus	GSM494290	PMID20188665
MECP2	RNA-Seq	WT sample. Diffrentially expressed genes (DEGs) of the contrast KO vs WT are displayed on the genome browser through blue bars (downregulated genes) and red bars (upregulated genes).	Cerebral cortex and Hippocampus/ 8 weeks	Mus musculus	GSM3666190	PMID31784358
MECP2	RNA-Seq	WT sample. Diffrentially expressed genes (DEGs) of the contrast KO vs WT are displayed on the genome browser through blue bars (downregulated genes) and red bars (upregulated genes).	Cerebral cortex and Hippocampus/ 8 weeks	Mus musculus	GSM3666191	PMID31784358

MECP2	RNA-Seq	WT sample. Diffrentially expressed genes (DEGs) of the contrast KO vs WT are displayed on the genome browser through blue bars (downregulated genes) and red bars (upregulated genes).	Cerebral cortex and Hippocampus/ 8 weeks	Mus musculus	GSM3666192	PMID31784358
MECP2	RNA-Seq	WT sample. Diffrentially expressed genes (DEGs) of the contrast KO vs WT are displayed on the genome browser through blue bars (downregulated genes) and red bars (upregulated genes).	Cerebral cortex and Hippocampus/ 8 weeks	Mus musculus	GSM3666193	PMID31784358
MECP2	RNA-Seq	WT sample. Diffrentially expressed genes (DEGs) of the contrast KO vs WT are displayed on the genome browser through blue bars (downregulated genes) and red bars (upregulated genes).	Cerebral cortex and Hippocampus/ 8 weeks	Mus musculus	GSM3666194	PMID31784358
MECP2	RNA-Seq	WT sample. Diffrentially expressed genes (DEGs) of the contrast KO vs WT are displayed on the genome browser through blue bars (downregulated genes) and red bars (upregulated genes).	Cerebral cortex and Hippocampus/ 8 weeks	Mus musculus	GSM3666195	PMID31784358
MECP2	RNA-Seq	WT sample. Diffrentially expressed genes (DEGs) of the contrast KO vs WT are displayed on the genome browser through blue bars (downregulated genes) and red bars (upregulated genes).	Cerebral cortex and Hippocampus/ 8 weeks	Mus musculus	GSM3666196	PMID31784358
MECP2	RNA-Seq	WT sample. Diffrentially expressed genes (DEGs) of the contrast KO vs WT are displayed on the genome browser through blue bars (downregulated genes) and red bars (upregulated genes).	Cerebral cortex and Hippocampus/ 8 weeks	Mus musculus	GSM3666197	PMID31784358
MECP2	RNA-Seq	WT sample. Diffrentially expressed genes (DEGs) of the contrast KO vs WT are displayed on the genome browser through blue bars (downregulated genes) and red bars (upregulated genes).	Cerebral cortex and Hippocampus/ 8 weeks	Mus musculus	GSM3666198	PMID31784358
MECP2	RNA-Seq	WT sample. Diffrentially expressed genes (DEGs) of the contrast KO vs WT are displayed on the genome browser through blue bars (downregulated genes) and red bars (upregulated genes).	Cerebral cortex and Hippocampus/ 8 weeks	Mus musculus	GSM3666199	PMID31784358
MECP2	RNA-Seq	KO sample. Diffrentially expressed genes (DEGs) of the contrast KO vs WT are displayed on the genome browser through blue bars (downregulated genes) and red bars (upregulated genes).	Cerebral cortex and Hippocampus/ 8 weeks	Mus musculus	GSM3666200	PMID31784358
MECP2	RNA-Seq	KO sample. Diffrentially expressed genes (DEGs) of the contrast KO vs WT are displayed on the genome browser through blue bars (downregulated genes) and red bars (upregulated genes).	Cerebral cortex and Hippocampus/ 8 weeks	Mus musculus	GSM3666201	PMID31784358

MECP2	RNA-Seq	KO sample. Diffrentially expressed genes (DEGs) of the contrast KO vs WT are displayed on the genome browser through blue bars (downregulated genes) and red bars (upregulated genes).	Cerebral cortex and Hippocampus/ 8 weeks	Mus musculus	GSM3666202	PMID31784358
MECP2	RNA-Seq	KO sample. Diffrentially expressed genes (DEGs) of the contrast KO vs WT are displayed on the genome browser through blue bars (downregulated genes) and red bars (upregulated genes).	Cerebral cortex and Hippocampus/ 8 weeks	Mus musculus	GSM3666203	PMID31784358
MECP2	RNA-Seq	KO sample. Diffrentially expressed genes (DEGs) of the contrast KO vs WT are displayed on the genome browser through blue bars (downregulated genes) and red bars (upregulated genes).	Cerebral cortex and Hippocampus/ 8 weeks	Mus musculus	GSM3666204	PMID31784358
MECP2	RNA-Seq	KO sample. Diffrentially expressed genes (DEGs) of the contrast KO vs WT are displayed on the genome browser through blue bars (downregulated genes) and red bars (upregulated genes).	Cerebral cortex and Hippocampus/ 8 weeks	Mus musculus	GSM3666205	PMID31784358
MECP2	RNA-Seq	KO sample. Diffrentially expressed genes (DEGs) of the contrast KO vs WT are displayed on the genome browser through blue bars (downregulated genes) and red bars (upregulated genes).	Cerebral cortex and Hippocampus/ 8 weeks	Mus musculus	GSM3666206	PMID31784358
MECP2	RNA-Seq	KO sample. Diffrentially expressed genes (DEGs) of the contrast KO vs WT are displayed on the genome browser through blue bars (downregulated genes) and red bars (upregulated genes).	Cerebral cortex and Hippocampus/ 8 weeks	Mus musculus	GSM3666207	PMID31784358
MECP2	RNA-Seq	KO sample. Diffrentially expressed genes (DEGs) of the contrast KO vs WT are displayed on the genome browser through blue bars (downregulated genes) and red bars (upregulated genes).	Cerebral cortex and Hippocampus/ 8 weeks	Mus musculus	GSM3666208	PMID31784358
MECP2	RNA-Seq	KO sample. Diffrentially expressed genes (DEGs) of the contrast KO vs WT are displayed on the genome browser through blue bars (downregulated genes) and red bars (upregulated genes).	Cerebral cortex and Hippocampus/ 8 weeks	Mus musculus	GSM3666209	PMID31784358
MECP2	HI-C	WT sample. The tracks are visualized by flatarcs that connect the various parts of the genome together.	Cerebral cortex and Hippocampus/ 8 weeks	Mus musculus	GSM4142678	PMID31784358
MECP2	HI-C	KO sample. The tracks are visualized by flatarcs that connect the various parts of the genome together.	Cerebral cortex and Hippocampus/ 8 weeks	Mus musculus	GSM4142681	PMID31784358

TFs	In silico predictions	This file contains CISBP TFBS binding predictions on Mus musculus GRCm38/mm10 genome. Displayed on genome browser with a blue bar. Names of transcription factors, on each bar, are visible when zooming in.	Different Tissues	Mus musculus	The MEME suite. Motif Database	PMID21330290
TFs	ChIP-Seq/ ChIP-Exo	ReMap is a large scale integrative analysis of DNA-binding experiments for Homo sapiens, Mus musculus, Drosophila melanogaster and Arabidopsis thaliana transcriptional regulators. The acronym “nr” stands for “non-redundant peaks”, referring to peaks identified by ChIP-Seq, ChIP-Exo, DAP-seq experiments. The trace is displayed on the genome browser by means of blue bars. Names of transcription factors, on each bar, are visible when zooming in.	Different Tissues	Mus musculus	ReMap2022	PMID34751401
CRMs	ChIP-Seq/ ChIP-Exo	ReMap is a large scale integrative analysis of DNA-binding experiments for Homo sapiens, Mus musculus, Drosophila melanogaster and Arabidopsis thaliana transcriptional regulators. The acronym “CRM” stands for “Cis-Regulatory Element”, identified by to ChIP-Seq, ChIP-Exo, DAP-Seq experiments. The track is displayed on the genome browser by means of bars of different colours. Names of transcription factors, on each bar, are visible when zooming in.	Different Tissues	Mus musculus	ReMap2022	PMID34751401
CpG islands	In silico predictions	This track identifies CpG islands on the genome.	Whole Genome	Mus musculus	UCSC Genome Browser	-
