# Supplementary material for: RettDb: the Rett syndrome omics database to navigate the Rett syndrome genomic landscape
Source: Database (Oxford). 2024 Oct 16;2024:baae109. doi: 10.1093/database/baae109 (PMC11482253; doi:10.1093/database/baae109)
Supplement: baae109_Supp [file baae109_supp.zip › baae109_supp/Sup_Tab2_Cillari.pdf]

Software	Version
**Bedtools**	v2.30.0
**BEDOPS**	v2.4.38
**Bowtie2**	v2.4.4
**DESeq2**	v1.36.0
**Fastqc**	v0.11.9
**Htseq-count**	v0.13.5
**Juicer**	v1.99
**Macs**	1.4.2 20120305
**Macs2**	v2.2.7.1
**Multiqc**	v1.10.1
**Samtools**	v1.12
**Trimgalore**	v0.6.6
**WashU genome browser**	v54.0.4

Supplementary Table 2. List of the software used for the analysis.
