# Supplementary material for: RettDb: the Rett syndrome omics database to navigate the Rett syndrome genomic landscape
Source: Database (Oxford). 2024 Oct 16;2024:baae109. doi: 10.1093/database/baae109 (PMC11482253; doi:10.1093/database/baae109)
Supplement: baae109_Supp [file baae109_supp.zip › baae109_supp/Tutorial_RettDb.pdf]

RettDb Tutorial

N. Cillari, G. Neri, N. Pisanti, P. Milazzo, U. Borello

Date of Creation: 2024-08-21

Version 1.0

This is a short step-by-step tutorial on the **RettDb** tool.

For a full description of the functionality of the genome browser, follow this **link** to the full documentation of the WashU genome browser on which RettDb is built.

1. Start by defining the Region of Interest

Select the gene or the genomic region of interest by clicking the `Genomic Region Locator` grey box.

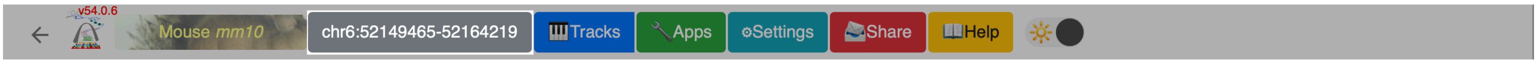

A dialog box opens with the following search options:

- Gene symbol;
- SNP ID (Single Nucleotide Polymorphism);
- The coordinates of the genomic region of interest.

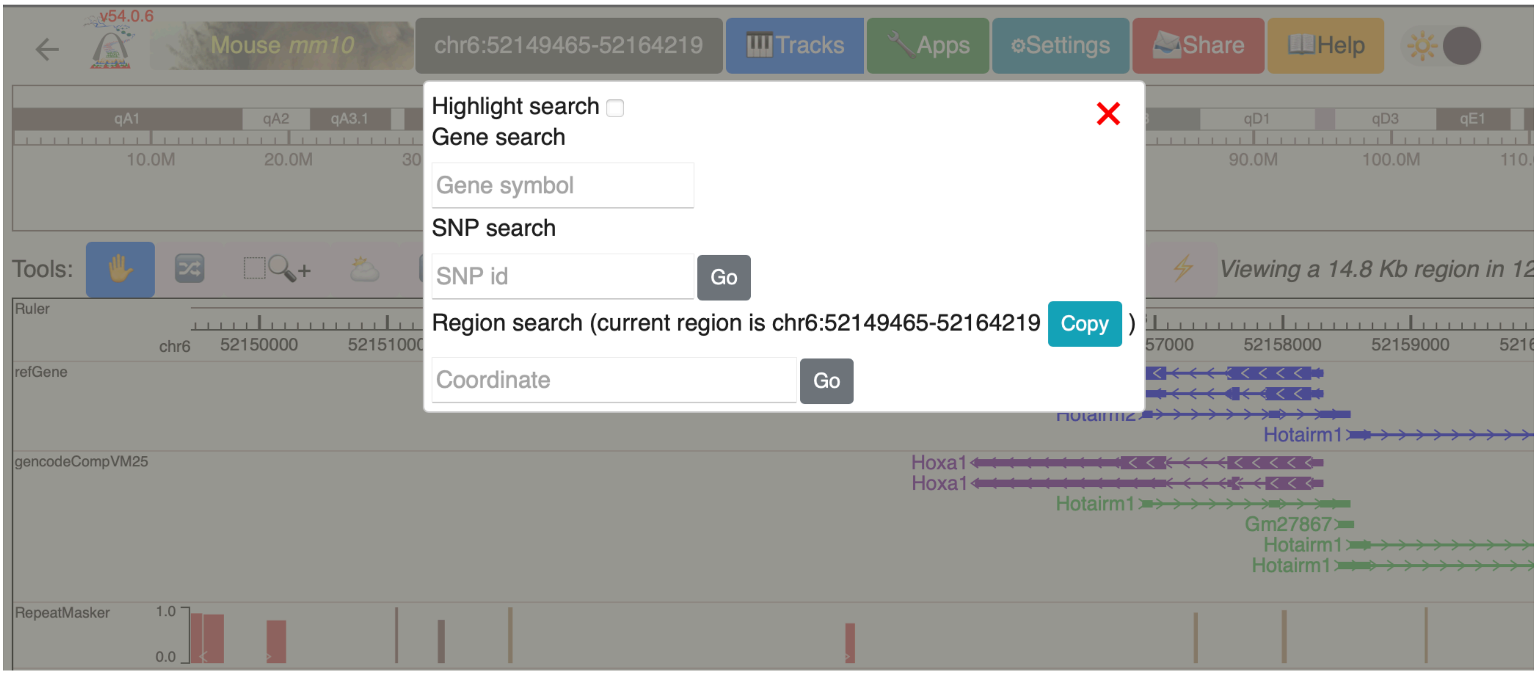

In this specific case, we search for the *Mus musculus* ***Pak3*** gene (Gene Symbol: *Pak3*; Full Name: p21 (RAC1) activated kinase 3; Gene ID: 18481).

Entering the gene name opens a dropdown menu showing the gene's splicing variants. To select a specific variant, click on it.

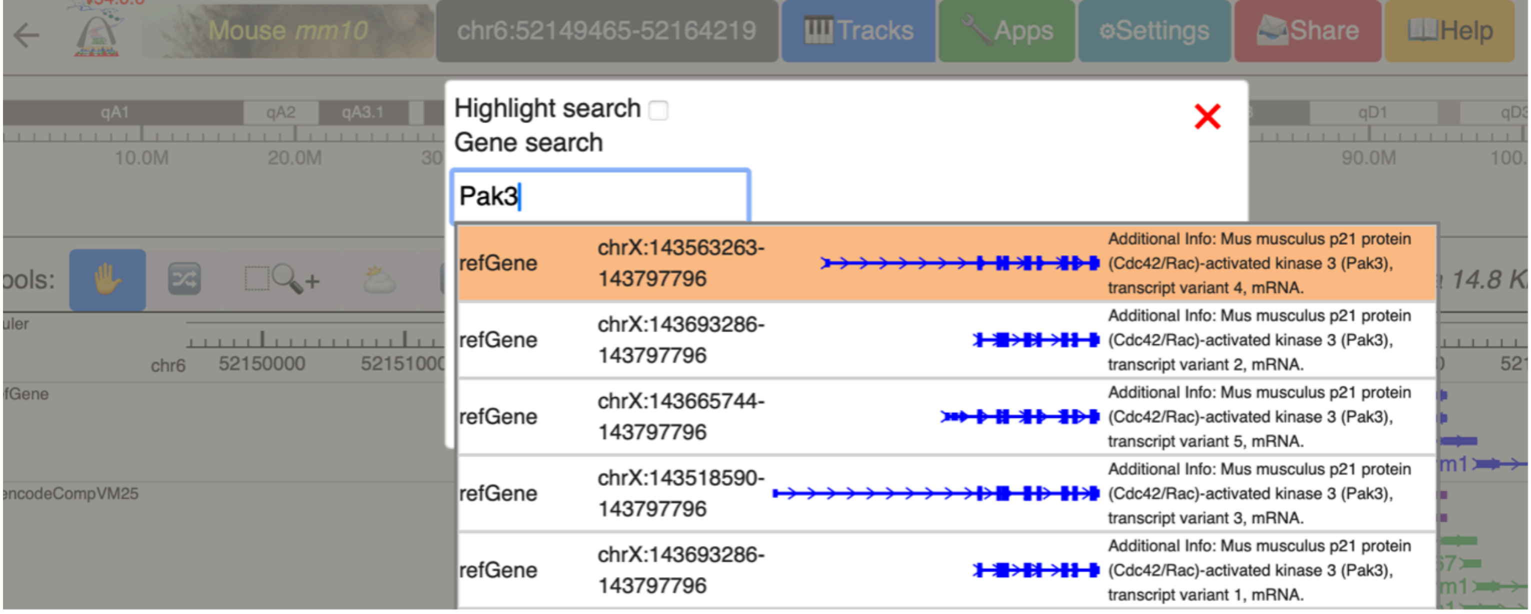

Ticking the `Highlight search` option highlights the gene of interest locus in the entire genome browser view.

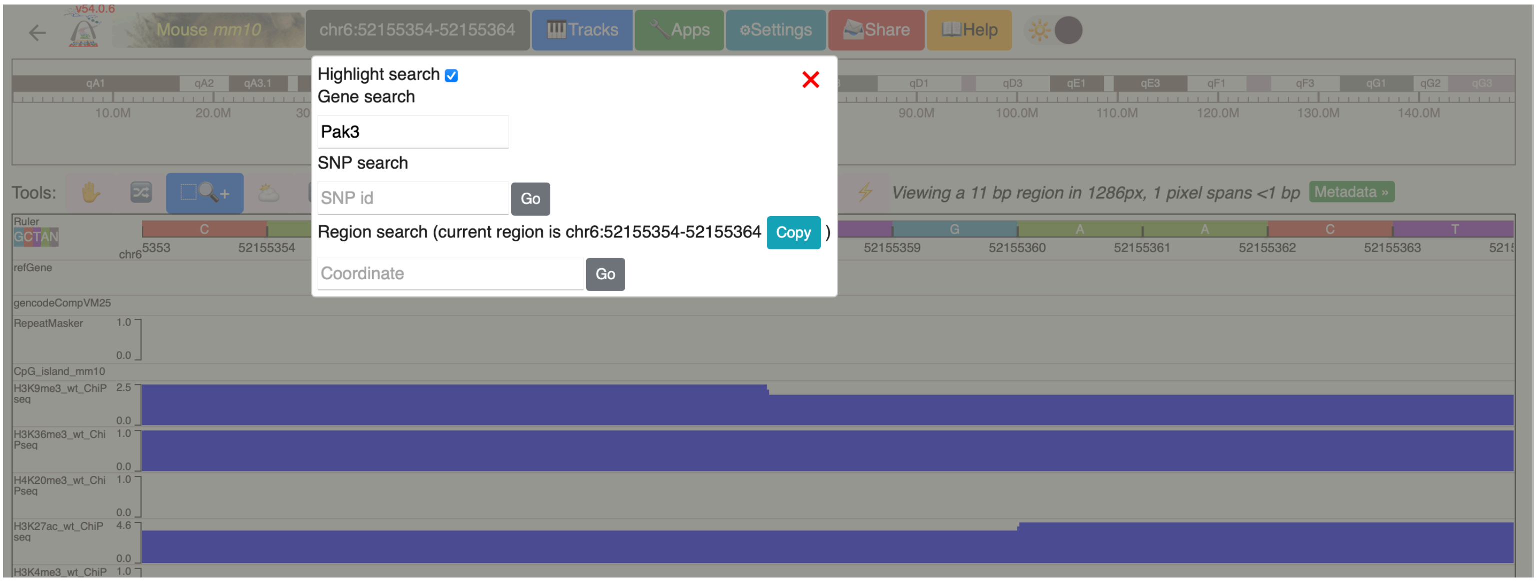

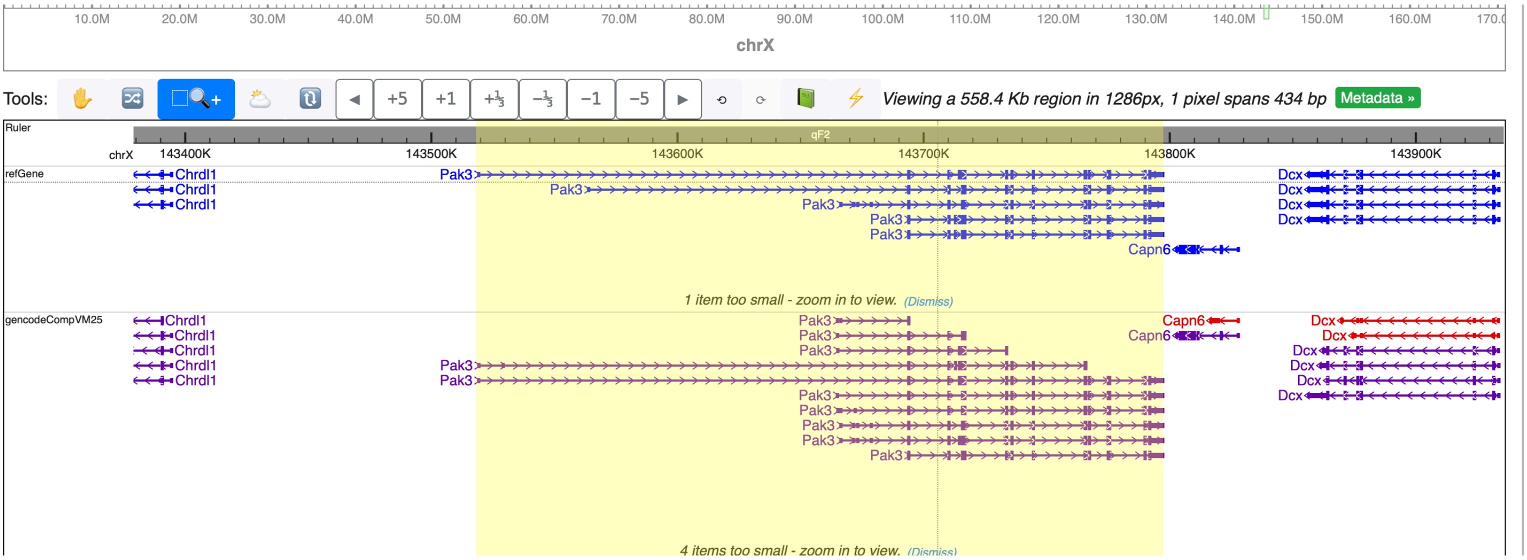

Alternatively, it is possible to search for a **SNP** ID or a specific **genomic region** by genomic coordinates.

1.1 Focus on the locus of interest

After selecting *Pak3*, the gene of interest, we land on the genomic region containing the gene locus.

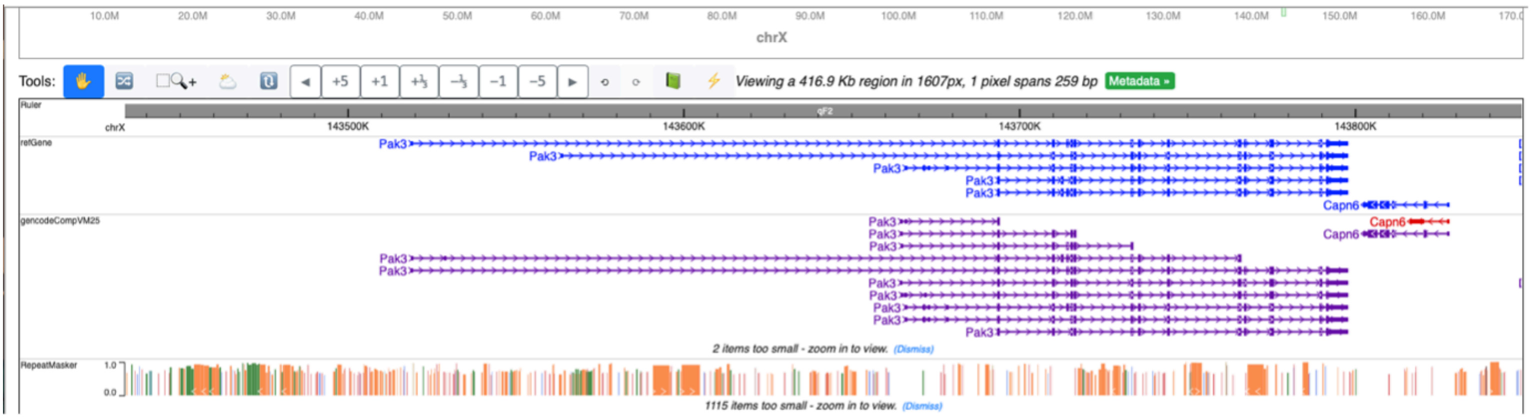

This view shows *Pak3* variants as described by the **RefGene** and the **Gencode** projects and the sequences with interspersed repeats and low complexity screened by the program **RepeatMasker**.

1.2 Refine the search using the Tools Bar

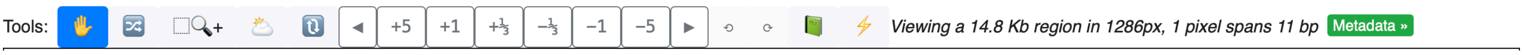

This bar allows access to different tools:

- Drag (Yellow Hand): drag and drop to move right or left from the current position
- Reorder (one or multiple tracks): change the order of the tracks
- Magnify: select a specific region to zoom in
- Zoom in and out on the genomic position
- Show history (Green Book)

Keyboard shortcuts to these functionalities are listed in the *Addenda* section.

1.3 Get Genomic Information

Clicking on each of the *Pak3* transcripts shows genomic information, regarding each variant, and a link to **NCBI gene** repository for further details.

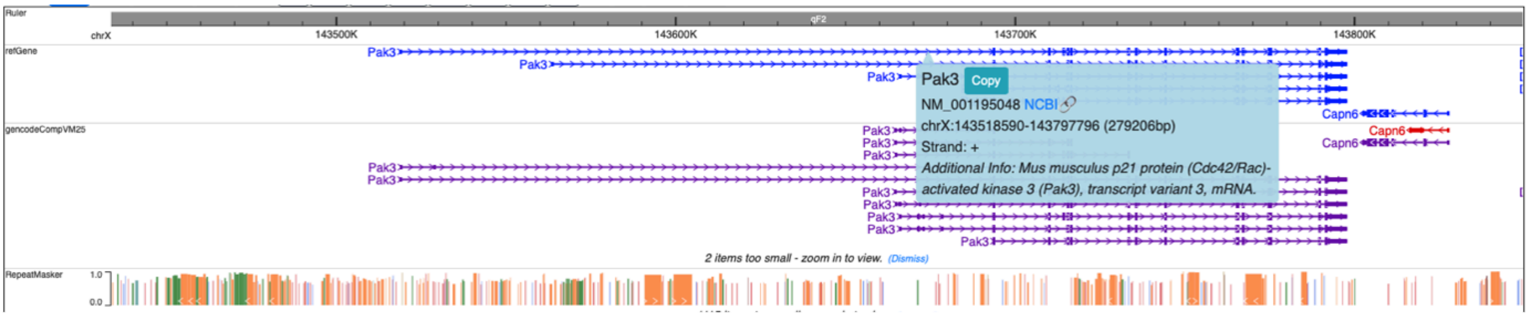

Clicking on a RepeatMasker signal bar provides information on the type of sequence recognized by the program as well as its genomic position and degree of divergence.

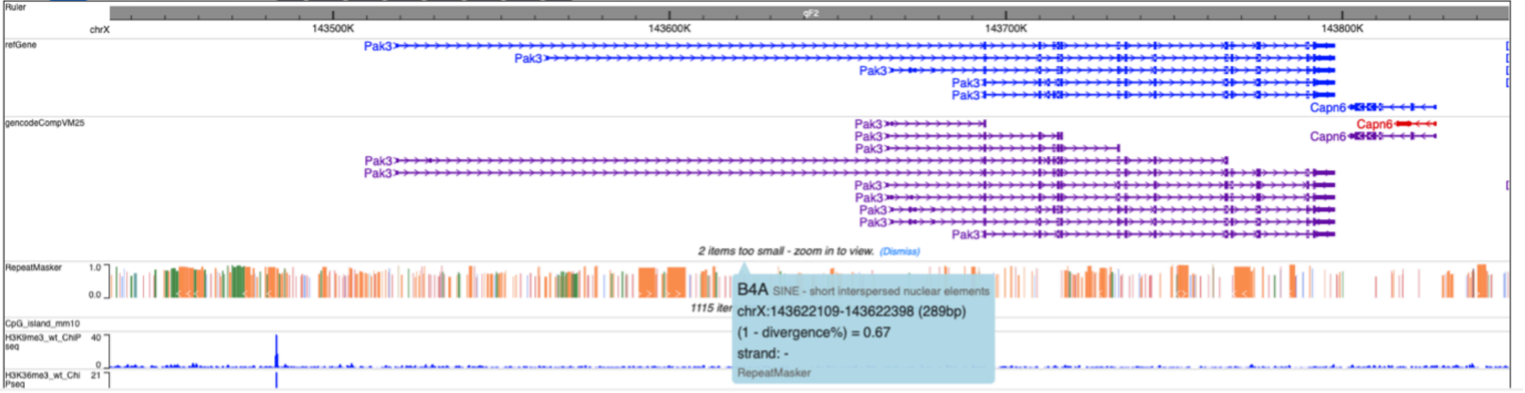

2. Browsing the Epigenomic Landscape

The transcriptional regulatory context of genome-wide MeCP2 binding activity is visualized below the RepeatMasker track.

2.1 Tracks Layout

Clicking on the blu `Tracks` box, a dropdown menu opens allowing to get the list of available tracks, to add or to remove tracks, and to change their orders at the user's convenience. Refer to the *Addenda* section for instructions to access short tutorials.

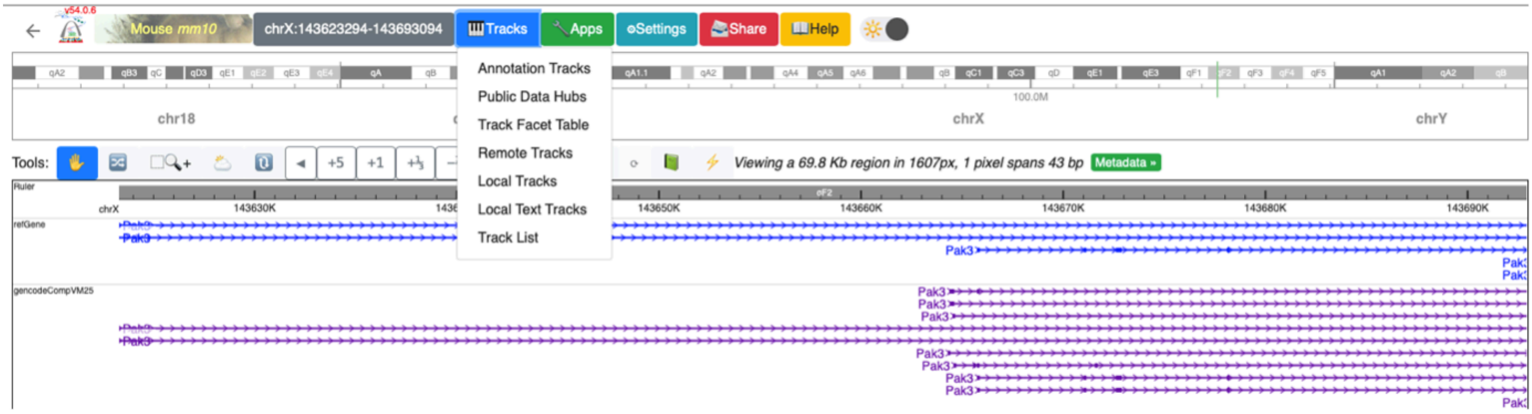

For other specific functionalities, refer to the **WashU Epigenome Browser full guide**

2.2 Analyzing MeCP2 binding activity

2.2.1 Genome-wide MeCP2 binding and Chromatin States

These ChIP-Seq tracks allow to identify MeCP2 binding sites on the region of interest as well as markers of active or repressed chromatin states.

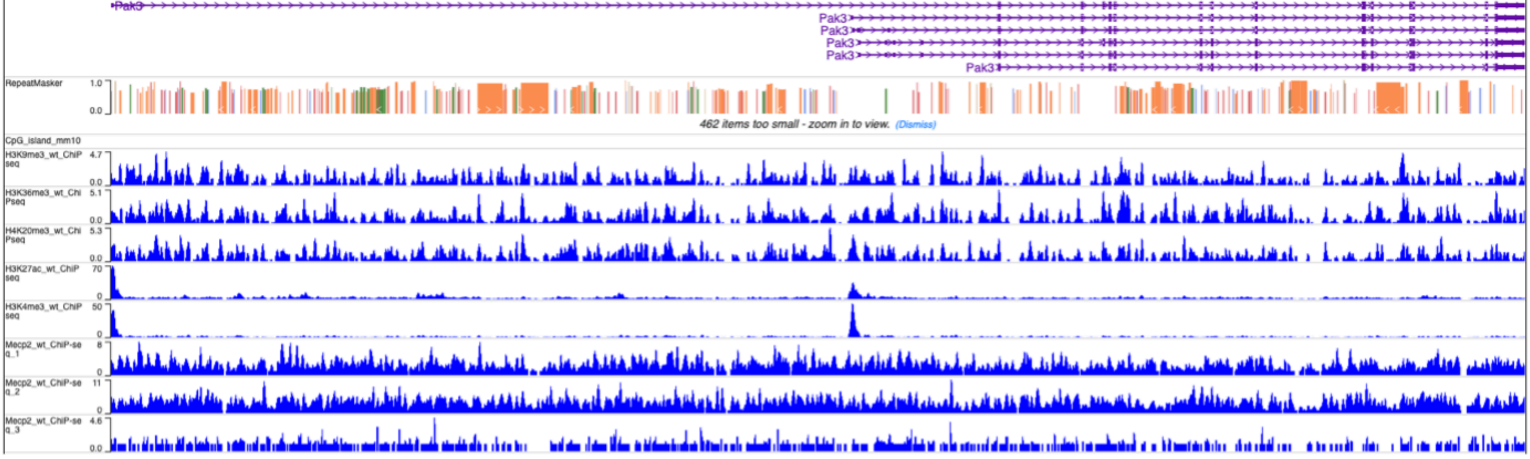

By clicking on the track name box a dropdown menu appears allowing to change different track parameters.

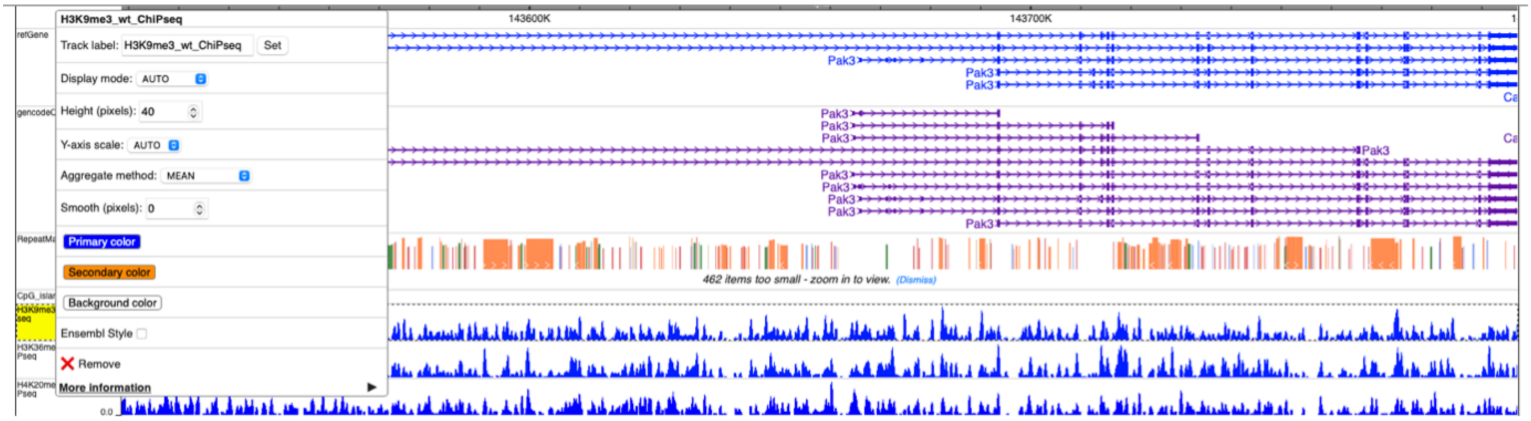

For further details on these parameters, refer to the **Track Customization** section of the **WashU Epigenome Browser** tutorial

2.2.2 MeCP2 regulatory elements

The ReMap and CISBP tracks allow to identify putative regulatory elements in the region of interest.

The NR (Non Redundant) and CRM (Cis Regulatory Modules) tracks of the **ReMap project** show putative enhancers as well as the transcription factors (TFs) binding to the different regulatory elements.

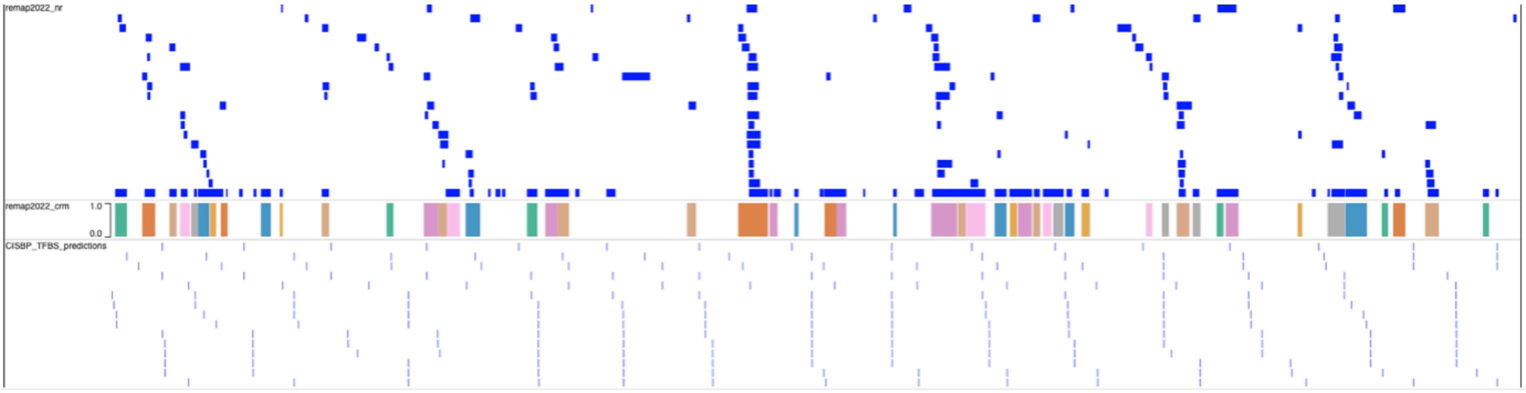

Clicking on the ReMap NR single boxes shows the TF symbols, the cell system in which this TF:DNA interaction was experimentally observed, as well as the genomic coordinates of the regulatory element.

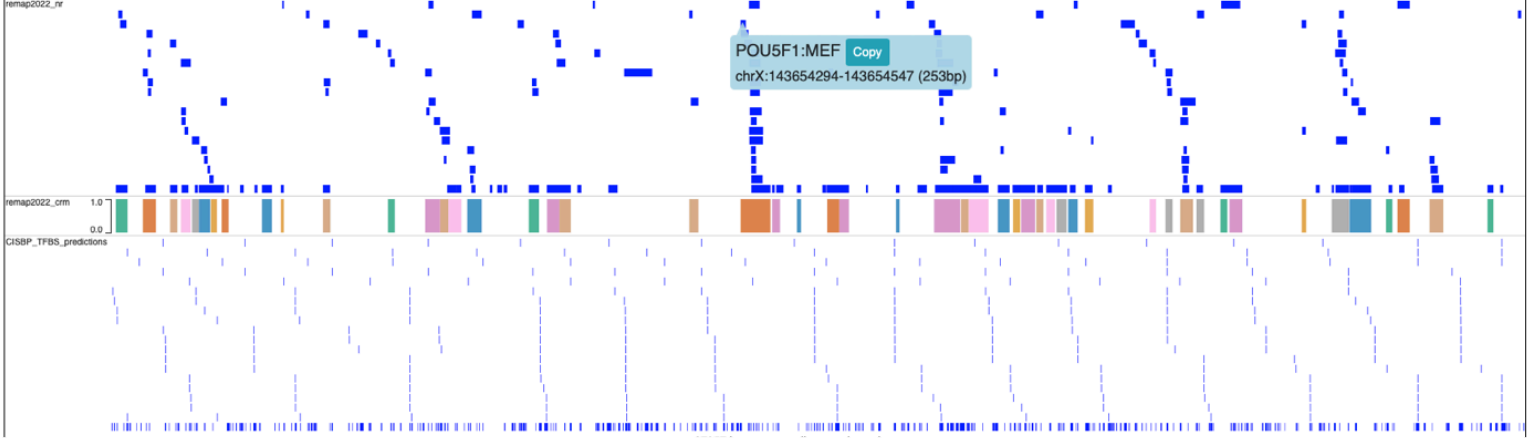

Similarly, clicking on the ReMap CRMs single boxes shows the first symbol of the list of TFs binding the Regulatory Module, the cell system in which this TF:DNA interaction was experimentally observed, as well as the genomic coordinates of the regulatory element.

To obtain the full list of the TFs binding on the selected module, the user shall use the selected module genomic coordinates to interrogate the ReMap CRM database.

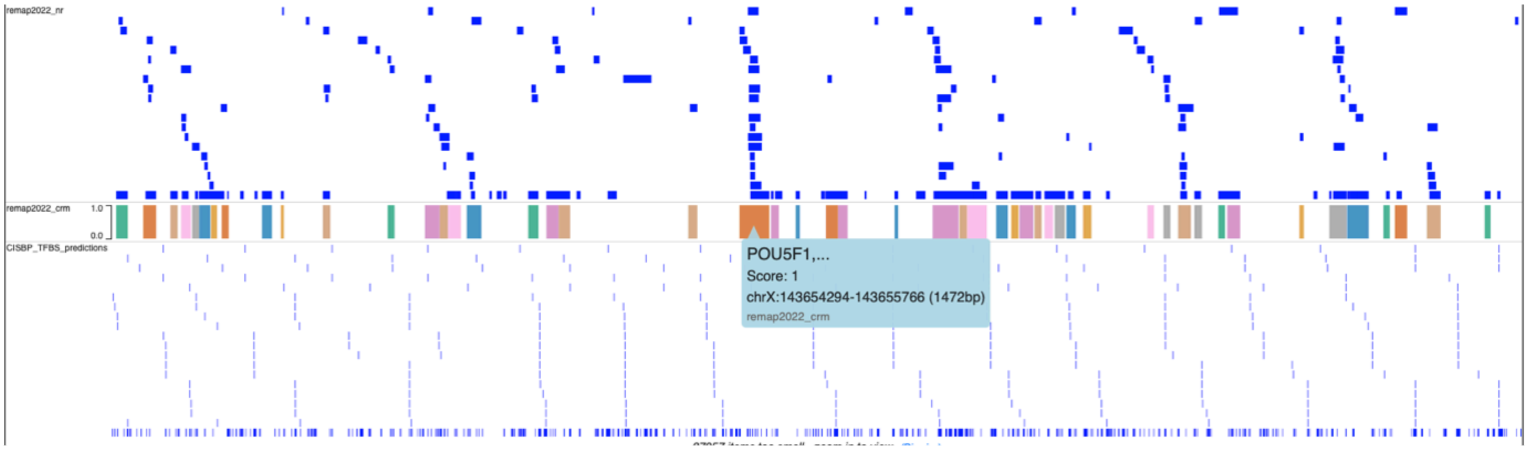

To identify predicted transcription factors binding sites, the user can browse the CISBP TF project prediction track.

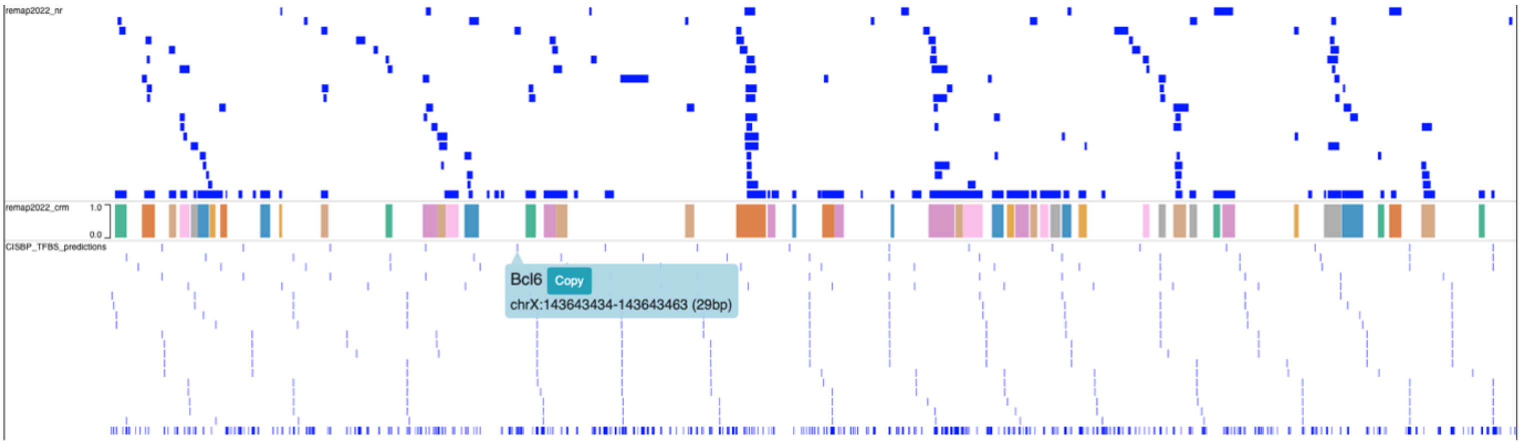

Comparing ReMap and CISBP tracks with the ChIP-Seq peaks, it is possible to identify to which putative enhancers MeCP2 binds and which TFs are putative MeCP2 co-regulators.

2.2.3 MeCP2 Target Genes

To assess if the gene of interest is regulated by MeCP2, we provide a track showing differentially expressed genes derived from RNA-Seq analysis on Mecp2 KO vs WT mutant mice. The down-regulated genes are identified by a red box and the up-regulated ones by a blue box.

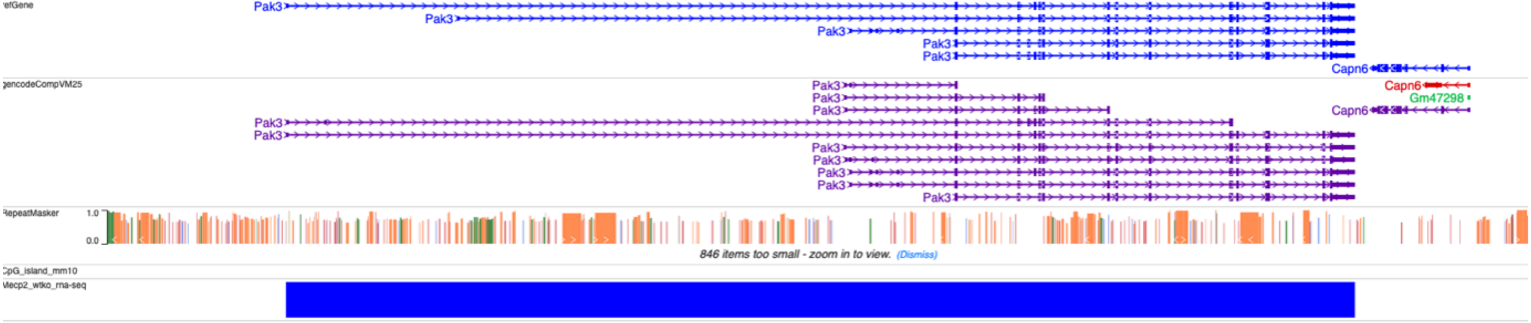

The presence of MeCP2 binding signal (MeCP2 ChIP-Seq tracks) on the loci of the differentially expressed genes or their regulatory elements allows to distinguish between a direct and indirect MeCP2 target gene.

2.2.4 Genomic Regulatory Modules Gene Interactions with Gene of Interest

HI-C tracks allows to identify the putative regulatory elements modulating the expression of the gene of interest. Those tracks show the interactions of different long-distance genomic regions containing the putative regulatory elements and the promoter of gene of interest.

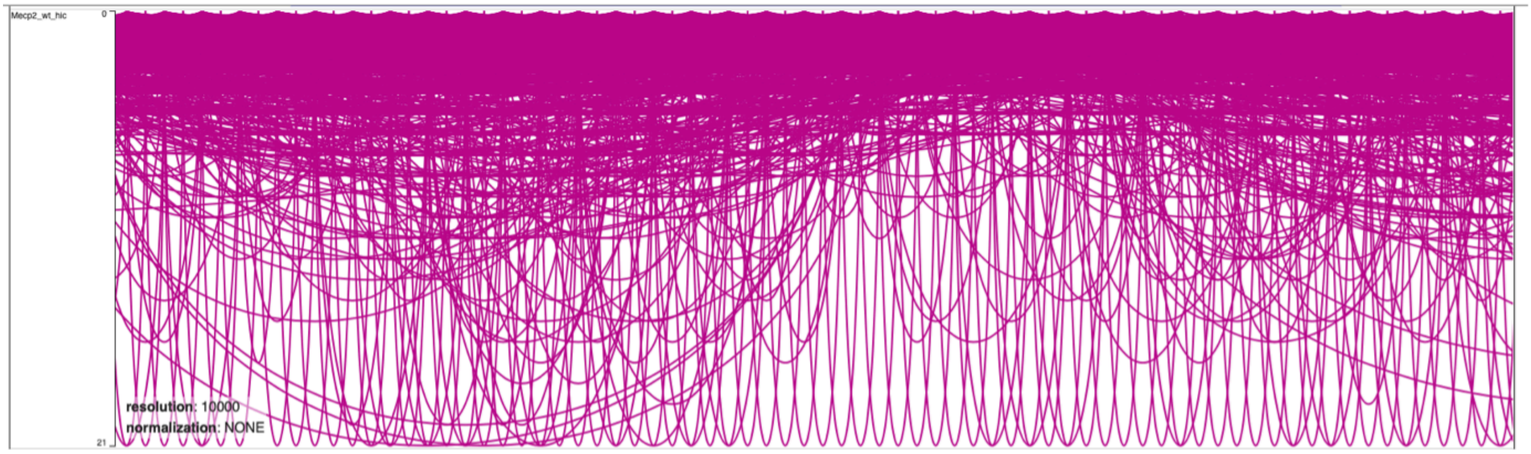

Chromatin interaction track visualization could be challenging to analyze. By right-clicking on the tracks box names a dropdown menu allows to change the visualisation parameters of the HI-C tracks.

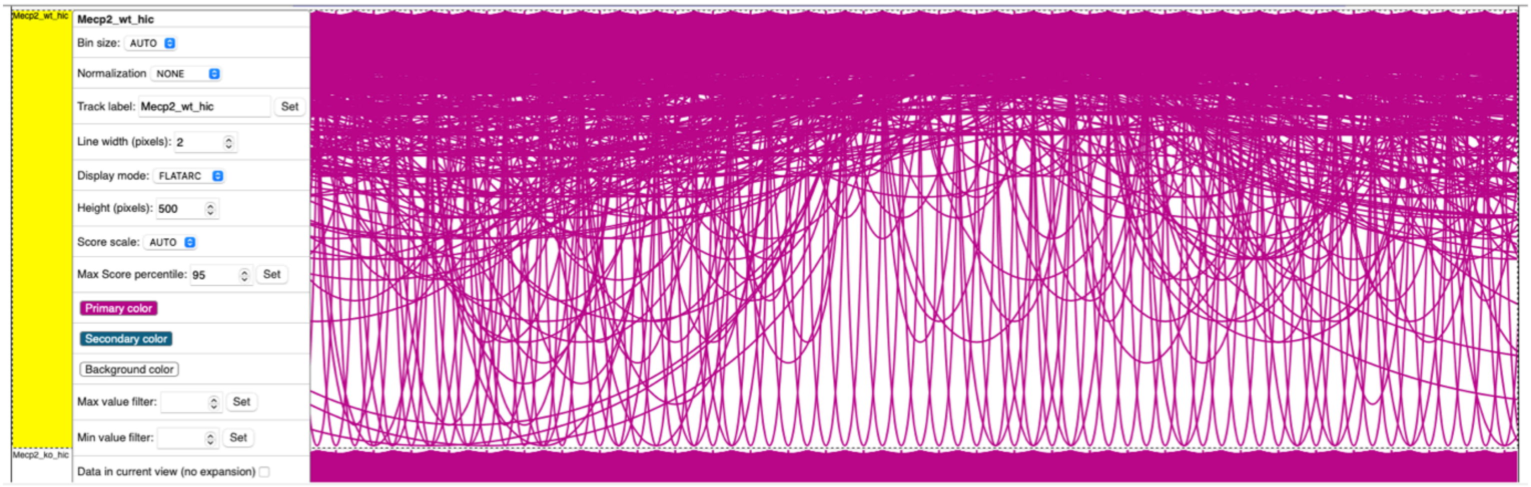

Modifying the `Bin Size`, `Score scale`, and `Data in current view (no expansion)` parameters it is possible to obtain a simplified track showing only chromatin loops of interest.

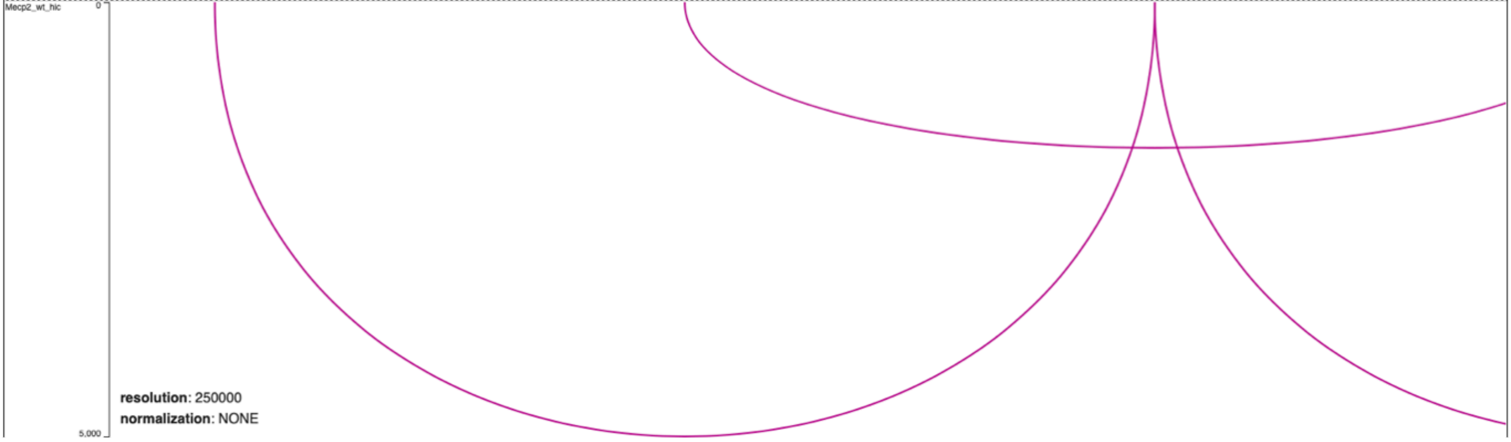

3. Addenda

3.1 Access to short tutorials

In the top panel above the genome browser, four icons summarize the basic functionality of the browser.

Clicking on `Add local tracks` allows the users to display a visual instruction to add tracks in local mode. Clicking on `Remove tracks` allows the users to display a visual instruction to remove unnecessary tracks. Clicking on `Re-order tracks` allows the users to display a visual instruction to change the order in which the tracks are displayed. Clicking on `Gene Search` allows the users to display a visual instruction to browse a specific gene of interest.

Add local tracks **Remove tracks** **Re-order tracks** **Gene Search**

3.2 Keyboard Shortcut

Windows	MacOS	Tool
Alt + D	⌥ + D	Drag
Alt + R	⌥ + R	Re-order
Alt + M	⌥ + M	Magnify
Alt + Z or X	⌥ + Z or X	Move Left or Right
